# Pluripotency-State-Dependent Role of Dax1 in Embryonic Stem Cells Self-Renewal

**DOI:** 10.1155/2021/5522723

**Published:** 2021-07-10

**Authors:** Jianrong He, Yuda Cheng, Yan Ruan, Jiali Wang, Yanping Tian, Jiaqi Wang, Fengsheng Wang, Chen Zhang, Yixiao Xu, Lianlian Liu, Meng Yu, Jiangjun Wang, Binyu Zhao, Yue Zhang, Yi Yang, Gaoke Liu, Wei Wu, Ping He, Jiaxiang Xiong, He Huang, Junlei Zhang, Rui Jian

**Affiliations:** ^1^Department of Anesthesiology, The Second Affiliated Hospital, Chongqing Medical University, Chongqing 400010, China; ^2^Laboratory of Stem Cell & Developmental Biology, Department of Histology and Embryology, Army Medical University, Chongqing 400038, China; ^3^Southwest Hospital/Southwest Eye Hospital, The First Hospital Affiliated to Army Medical University, Chongqing 400038, China; ^4^Experimental Center of Basic Medicine, College of Basic Medical Sciences, Army Medical University, Chongqing 400038, China; ^5^Thoracic Surgery Department, Southwest Hospital, The First Hospital Affiliated to Army Medical University, Chongqing 400038, China; ^6^Cardiac Surgery Department, Southwest Hospital, The First Hospital Affiliated to Army Medical University, Chongqing 400038, China

## Abstract

Dax1(also known as Nr0b1) is regarded as an important component of the transcription factor network in mouse embryonic stem cells (ESCs). However, the role and the molecular mechanism of Dax1 in the maintenance of different pluripotency states are poorly understood. Here, we constructed a stable Dax1 knockout (KO) cell line using the CRISPR/Cas9 system to analyze the precise function of Dax1. We reported that 2i/LIF-ESCs had significantly lower Dax1 expression than LIF/serum-ESCs. Dax1KO ES cell lines could be established in 2i/LIF and their pluripotency was confirmed. In contrast, Dax1-null ESCs could not be continuously passaged in LIF/serum due to severe differentiation and apoptosis. In LIF/serum, the activities of the Core module and Myc module were significantly reduced, while the PRC2 module was activated after Dax1KO. The expression of most proapoptotic genes and lineage-commitment genes were drastically increased, while the downregulated expression of antiapoptotic genes and many pluripotency genes was observed. Our research on the pluripotent state-dependent role of Dax1 provides clues to understand the molecular regulation mechanism at different stages of early embryonic development.

## 1. Introduction

Dax1 (Dosage-sensitive sex reversal-adrenal hypoplasia congenital on the X-chromosome gene-1) has been suggested to play important roles in reproductive development, sex determination, steroidogenesis, and tumorigenesis. Recently, Dax1 is identified as a core member of pluripotency gene regulatory network [1, 2]. Overexpression of Dax1 supports LIF-independent self-renewal in mouse ESCs [3], while knockdown of Dax1 by siRNA/shRNA or knockout of Dax1 using the Cre-lox system induces differentiation of ESCs [2–7]. Dax1 can inhibit extraembryonic endoderm differentiation by binding to the promoter of Gata6 and inhibiting its transcription and can inhibit trophectoderm differentiation either independently of or cooperatively with Oct4 [3]. It is proposed that Dax1 and Nanog act in parallel to maintain an optimal pluripotent state [3]. Additionally, two independent reports confirmed that Dax1 is necessary for pluripotency inducing [3, 8]. However, all the investigations concerning the role of Dax1 on pluripotency maintenance were carried out in ESCs cultured with serum and LIF (Supplementary Table 1).

The various culture conditions have allowed the capture of different pluripotency states of mouse ESCs in vitro. ESCs grown in the presence of serum and LIF were referred to as “conventional” ESCs, which include all stages of ESCs from naive to formative and/or primed state. Therefore, the conventional ESCs exhibit heterogeneity and metastability [9]. Recent studies have shown that ESCs can also be maintained in serum-free N2B27 medium supplemented with two small molecule inhibitors (2i), the GSK3 inhibitor CHIR99021, and MEK inhibitor PD0325901. 2i/LIF-ESCs appear to be homogeneous and have different gene expression profile and epigenome from those of conventional ESCs, which are postulated to represent the ground state of pluripotency [10]. Since the different pluripotency states are characterized and maintained by distinct transcriptional networks, each transcriptional factor may preferentially sustain a distinct pluripotency state [11, 12]. However, only a few transcription factors (such as Klf2 and Myc) have been compared of their functional characteristics under different pluripotency states. It remains elusive whether Dax1 has similar functions in ground state ESCs as in the conventional ESCs. And the effect of Dax1 withdrawal on the pluripotency-associated transcription factor network is not well characterized.

Here, we established a stable Dax1-knockout (KO) cell line and analyzed the effect of Dax1KO on the cell phenotypes and the pluripotency gene regulatory network under LIF/serum and 2i/LIF conditions, respectively. Our results revealed that the role of Dax1 is dependent on the pluripotency state.

## 2. Results

### 2.1. Expression Pattern Analysis of Dax1 in Mouse ESCs

**Figure 1:**
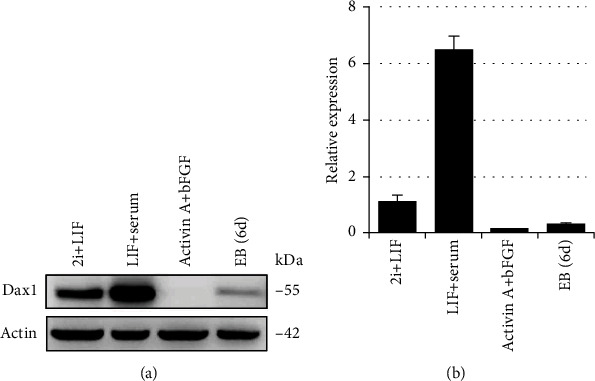
The expression level of Dax1 in 2i/LIF-ESCs is much lower than that in LIF/serum-ESCs. (a) Western blot analysis shows the expression of Dax1 in ESCs in different culture conditions. (b) Quantitative real-time PCR analysis shows the transcription level of Dax1 in different culture conditions. Data are the averages of biological triplicates ± standard error of the mean (SEM).

A publicly available RNA-seq assay of the first days of embryonic development indicated that Dax1 expression peaked in the ICM of E4.0 (Figure S1). Consistently, Dax1 is expressed at a high level in undifferentiated ESCs, but not in epiblast stem cells (EpiSCs) and differentiated ESCs [13]. Here, we investigated whether the expression of Dax1 is affected by the various culture conditions of ESCs. Western blot revealed that the Dax1 protein level in ESCs in 2i/LIF was significantly lower than that in LIF/serum (Figure 1(a)). The transcriptional level of Dax1 was further validated by qRT-PCR. ESCs in LIF/serum condition expressed the highest Dax1 mRNA, whereas ESCs in 2i/LIF expressed it at a level 6.4-fold lower (Figure 1(b)). Dax1 protein and mRNA level were either undetectable or expressed in very low concentrations in the samples of EpiSCs and embryoid bodies (EB). These results suggested that the expression of Dax1 is affected by different stages of pluripotency, and the decrease of Dax1 during ground state pluripotency is regulated at the transcriptional level.

### 2.2. Establishment of Dax1KO ES Cell Line under the 2i/LIF Condition

**Figure 2:**
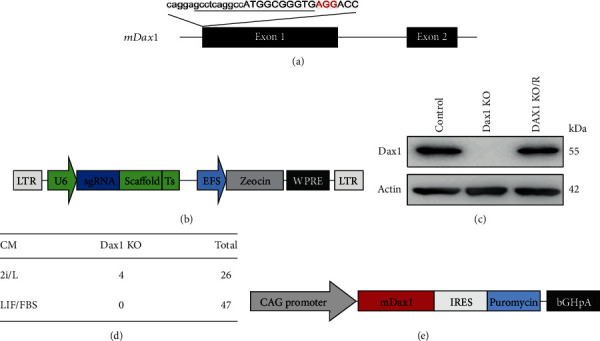
Establishment of Dax1KO-ESCs in 2i culture. (a) Schematic of the Cas9/sgRNA target site in the mouse Dax1 genomic locus. The sgRNA-targeting sequence is underlined, and the PAM sequences are marked in red. (b) Schematic diagram of lentivirus-based dual U6-sgRNA expression cassette constructs with the cassette of EFS-zeocin. (c) The expression of Dax1 and Actin was detected by Western blotting. (d) Dax1 cDNA was cloned into an expression vector carrying a CAG promoter and within the transcription unit linked to the puromycin resistance gene by an IRES. (e) Dax1-null colonies screened under LIF/serum and 2i conditions.

It was demonstrated that the knockout of Dax1 exon by the replacement with an antibiotic selection marker cassette failed to generate undifferentiated ESCs in LIF/serum [4]. However, a much lower expression level of Dax1 in 2i/LIF condition strongly suggests a nonessential role of Dax1 in ground state ESCs (Figure 1). To confirm this possibility and further analyze the functional role of the Dax1, CRISPR/Cas9 editing system was used to produce Dax1 knockout (KO) ES cell line in the two conditions (Figures 2(a) and 2(b)). After targeting, ESCs were plated at low density, and the Dax1 level of individual colony was verified by Western blotting analysis (Figure 2(c)). In 2i/LIF condition, 4 Dax1KO cell colonies were identified (Dax1KO-7, -10, -13, -23) from 26 colonies (Figure 2(d)), among which Dax1KO-7 was used in subsequent functional experiments. In LIF/serum, however, we did not obtain a Dax1KO cell colony although the expression of Dax1 was reduced in some colonies (Figure 2(d)). This result confirmed that Dax1 is necessary for ESCs survival in LIF/serum.

To rule out the possible off-target effects of Dax1KO, a rescue cell line was generated by introducing the Dax1 expression vector into the Dax1KO-null cells (Figure 2(e)). We identified a cell colony, named Dax1KO/R, in which the Dax1 expression level was close to that of the control (Figure 2(c)).

### 2.3. Dax1 Is Essential for Self-Renewal of Conventional ESCs but Is Dispensable for 2i/LIF-ESCs

**Figure 3:**
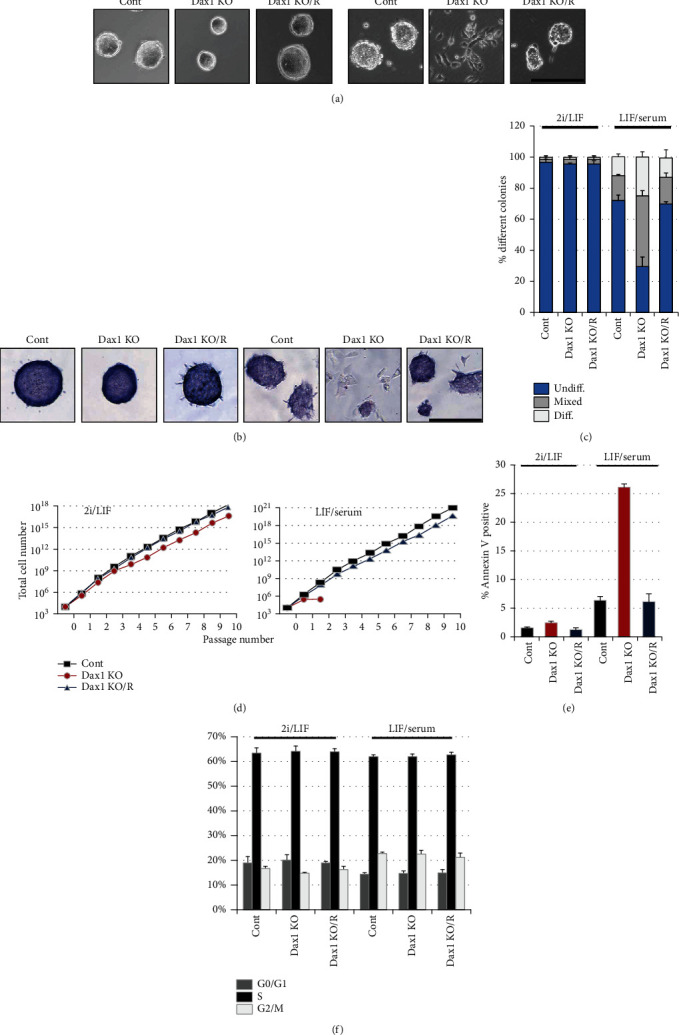
Properties of Dax1KO-ESCs in 2i/LIF and LIF/serum culture. (a) Morphology of colonies formed by the indicated cell lines. Cells were grown in 2i/LIF or LIF/serum for 5 days. Scale bar, 100 *μ*m. (b) AP staining of colonies formed by the indicated cell lines. Cells were grown in 2i/LIF or LIF/serum for 5 days. Scale bar, 100 *μ*m. (c) Percentage of colony types formed by cells is shown. Diff.: differentiated; Undiff.: undifferentiated. (d) Total cell number of the indicated lines cultured for multiple passages. (e) Cell cycle profiling of the indicated cell lines by FACS with propidium iodide (PI) staining and proportions of the cells at each cell cycle were shown in the graph. (f) The apoptosis rate of Dax1KO-ESCs cultured in the indicated conditions. Exponentially growing cells were analyzed for apoptosis using Annexin-V and propidium iodide. Data in (c)–(f) are represented as mean ± s.d.; *n* = 3.

To compare the functional capacity of Dax1 in different states of pluripotency, basic characterization was performed in Dax1KO-ESCs under 2i/LIF and LIF/serum conditions. In 2i/LIF, colony formation assays showed that the shape and number of colonies in Dax1KO cells were the same as control cells, but the size was a little smaller (Figures 3(a)–3(c)). Long-term propagation assay revealed that Dax1KO cells could be continuously passaged over four weeks although the growth rate slightly declined (Figure 3(d)). Consistently, fluorescence-activated cell sorting analysis (FACS) showed that the apoptosis rate of Dax1KO cells was just slightly increased (Figure 3(e)).

In contrast, Dax1KO cells presented a differentiated phenotype with massive cell death when transferred from 2i/LIF to LIF/serum condition (Figure 3(a)). The colonies formed by Dax1KO cells were even smaller and the percentage of AP-positive colonies (undifferentiated colonies) decreased significantly (Figures 3(b) and 3(c)). Dax1KO cells could not be passaged continuously in LIF/serum medium (Figure 3(d)). The apoptotic percentage of Dax1KO cells, even the first passage transferred from 2i/LIF, was strikingly exceeded up to 25% (Figure 3(e)). Previous studies showed that Dax1 knockdown caused a significant accumulation of Ewing's tumor cells in the G1 phase of the cell cycle [14]. However, our results showed that the proportion of the cells at the G1 phase was not altered (Figure 3(f)), which is in consist with another previous data [7].

As expected, such lethality of Dax1KO was completely rescued by the reexpression of Dax1 (Dax1KO/R), which indicates the gene-specific effects of Dax1 (Figures 3(a)–3(f)). Taken together, our results showed that Dax1 is dispensable for the self-renewal of ground state ESCs, but Dax1KO cells are highly vulnerable to differentiation and apoptosis in conventional culture condition.

### 2.4. Dax1KO Caused More Drastic Global Transcriptional Changes in Conventional ESCs than in Ground State ESCs

**Figure 4:**
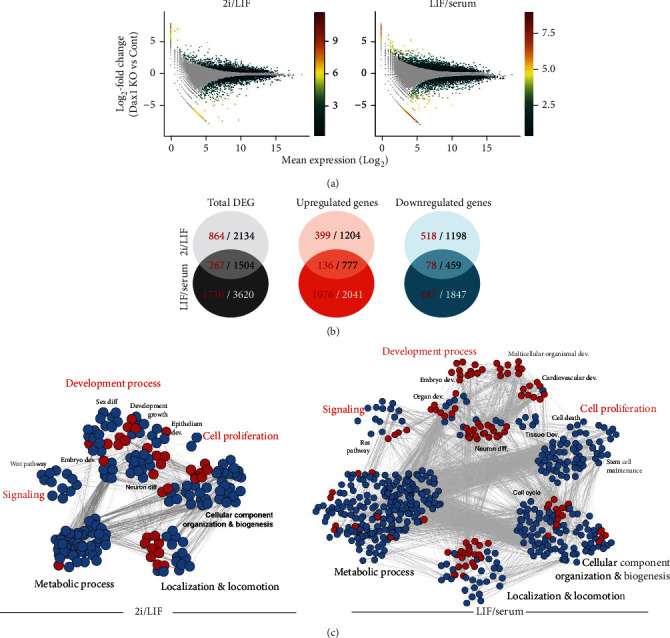
The global transcription profiles of Dax1KO cells in 2i/LIF and LIF/serum culture. (a) Scatterplots showing differentially expressed genes (DEGs) from Dax1 KO compared to wild-type ESCs cultured in 2i/LIF (left) and LIF/serum (right) medium. Grey dots are unaltered genes. Colored dots are significantly up-/downregulated genes in Dax1 KO cells with DESeq2 p (adj) ≤0.05, absolute value of fold change ≥1.2, the color of the dot correspond to the absolute value of fold change (red is high and blue is low). (b) Venn diagrams represent overlapped total DEGs (left), upregulated genes (middle), or downregulated genes (right) in Dax1 KO cells between 2i/LIF and LIF/serum medium. The red letters indicate the number of the DEGs with absolute fold change ≥2, and the black/white letters indicate the number of the DEGs with absolute fold change ≥1.2. (c) Enrichment map networks of GO terms corresponding to DEGs with absolute fold change ≥1.2 (blue) or DEGs with absolute fold change ≥2 (red) in Dax1 KO versus wild-type mESCs cultured in 2i/LIF (left) and LIF/serum (right) medium. Gene Ontology was analyzed by g: profiler, and visualized by the cytoscape plug-in: enrichment map. Nodes represent GO terms; edges connect nodes that share common genes.

To determine the mechanism underlying the different phenotypes of Dax1KO in different pluripotency states, we compared the global transcription profiles of Dax1KO cells under 2i/LIF and LIF/serum by RNA-seq analysis. Scatter plots showed differentially expressed genes (DEGs) of Dax1KO cells compared to wild-type ESCs (Figure 4(a), Supplementary Table 3). The overlapping DEGs in Dax1KO cells between the two conditions were shown in the Venn diagram (Figure 4(b), Figure S4a). Functional categorization of the DEGs was done by generating enrichment map networks of Gene Ontology (GO) terms (Figure 4(c), Supplementary Table 4).

In 2i/LIF, a total of 3638 DEGs was found in Dax1KO cells compared to the control (>1.2 fold, *P* < 0.05), of which 1981 genes and 1657 genes were up- and downregulated, respectively. GO analysis demonstrated the DEGs were significantly enriched in 173 gene sets including “Development process”, “Cell proliferation”, “signaling pathway”, “metabolic process”, “cellular component organization or biogenesis”, and “localization and locomotion” (>1.2 fold, *P* < 0.05). Then, we used more stringent filtering criteria of fold change >2 and FDR *P* value < 0.05 to identify the different expressed genes. The total number of DEGs in 2i/LIF was 1131, of which 535 genes and 596 genes were up- and downregulated, respectively. The DEGs were enriched in only 34 gene sets belongs to “Development process”, “cellular component organization or biogenesis” and “localization and locomotion” (the red point in Figure 4(c)). This may indicate that most DEGs related to self-renewal caused by Dax1KO in 2i/LIF do not undergo drastic changes (<2 fold).

In LIF/serum, the number of DEGs was much higher than that in 2i/LIF. There are 5124 DEGs (>1.2 fold, *P* < 0.05), of which 2818 genes were upregulated, 2306 genes were downregulated and 1977 DEGs (>2 fold, *P* < 0.05), of which 1212 genes were upregulated and 765 genes were downregulated, respectively. GO analysis demonstrated the DEGs were significantly enriched in 422 and 113 gene sets (>1.2 fold or 2 fold, respectively), much more than that in 2i/LIF. Consistent with more severe differentiation phenotypes of Dax1KO cells, the DEGs were significantly enriched in “Development process”, including “Neuron differentiation”, “Embryo development”, “Cardiovascular system development”, “Organ development”, “Multicellular organismal development”, and “Mammary gland lobule development” (>2 fold, *P* < 0.05). There were only 267 overlapping DEGs in Dax1KO cells under the two culture conditions, of which 136 shared upregulated genes and 78 shared downregulated genes (>2 fold, *P* < 0.05).

### 2.5. The Impact of Dax1KO on Transcription Networks (Modules) in Different Pluripotency States

Genome-wide transcriptional profiling showed that pluripotency regulatory network can be grouped into three relatively independent modules: modules for the core pluripotency factors (Core module), the Polycomb complex factors (PRC module), and the Myc-related factors (Myc module) [15] in conventional ESCs. While transcriptome comparisons show that the core module is likely to be equally important in 2i ESCs, 2i ESCs are unaffected by the low Myc expression levels and the functions of PRC2 may be redundant in 2i ESCs [16, 17].

**Figure 5:**
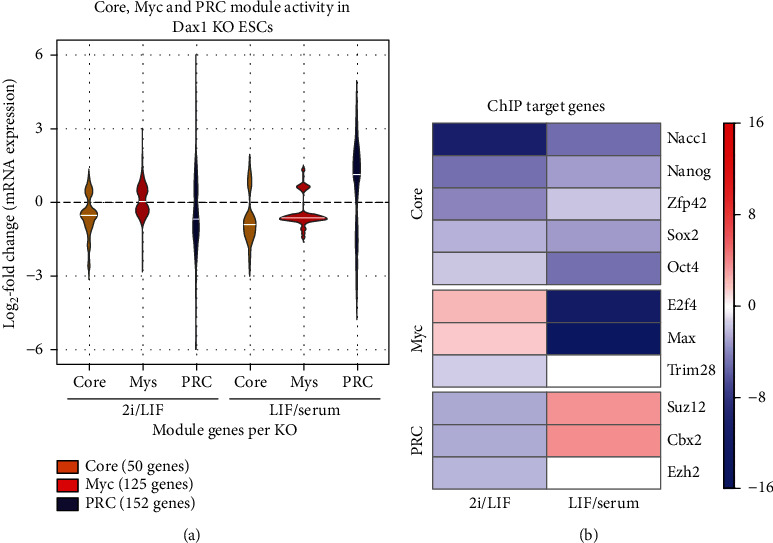
The impact of Dax1KO on signaling pathways and transcription networks (modules) in different pluripotency states. (a) A violin plot representing changes in CORE, MYC, and PRC module gene expression in Dax1 KO compared to wild-type mESCs cultured in 2i/LIF and LIF/serum medium. (b) Heatmap showing the geometric *P* values calculated by gene set overlap analysis between gene sets from ChIP-seq and DEGs (Dax1 KO compared to wild-type mESCs cultured in 2i/LIF and LIF/serum). The red cells correspond to upregulated DEGs; blue cells correspond to downregulated DEGs. Color intensity is proportional to log10 (*P* value).

The impacts of Dax1KO on these three modules were analyzed under LIF/serum and 2i/LIF conditions. After Dax1KO, the activity of Core module was reduced under the both culture conditions (Figure 5(a)). And downregulated genes of Dax1KO cells were highly correlated with the ChIP-target genes of Core module members (Figure 5(b)). The loss of Dax1 expression leads to a decline in Myc module activities and downregulated genes of Dax1KO cells were correlated with the ChIP-target genes of Myc module members under LIF/serum conditions (Figures 5(a) and 5(b)). The activity of PRC module increased under LIF/serum conditions but decreased in 2i/LIF (Figure 5(a)). Consistently, the upregulated and downregulated genes of Dax1KO cells were correlated with the ChIP-target genes of PRC2 in LIF/serum and 2i/LIF, respectively (Figure 5(b)). Together, it is suggested that the impact of Dax1KO on these ESC modules is dependent on the pluripotency state.

### 2.6. Validating the Expression of Self-Renewal-Related Genes after Dax1KO

**Figure 6:**
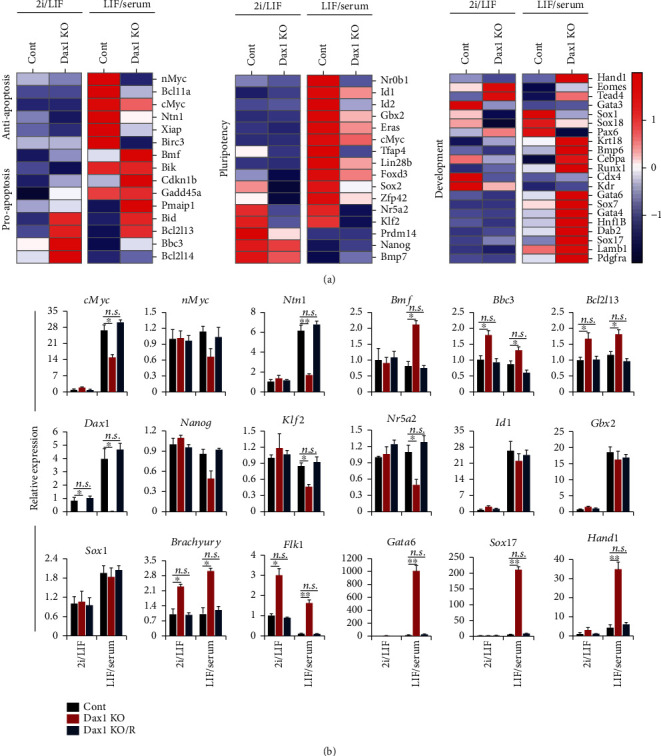
Expression of self-renewal-related genes after Dax1KO in different pluripotency states. (a) Heatmap of the apoptosis-related genes, pluripotency-associated genes, and lineage commitment genes after Dax1KO in 2i/LIF and LIF/serum. (b) qRT-PCR analysis of the indicated genes. Data are normalized to Gapdh and shown relative to WT ESCs (set at 1.0). Data are represented as mean ± SD; *n* = 3. ^∗^*P* ≤ 0.05; ^∗∗^*P* ≤ 0.01. All *P* values were calculated using Student's *t*-test.

The effect of Dax1KO on the pluripotency-associated transcription factor network prompted us to further analyze the changes in the expression of individual genes induced by Dax1KO. In 2i/LIF, the expressions of proapoptotic genes were slightly increased, while the expression of antiapoptotic genes, most pluripotency-related genes, and endoderm markers had no significant change. And no consistent changes in other lineage commitment genes were observed (Figure 6(a)). In LIF/serum, the expression of most proapoptotic genes was drastically increased, while the antiapoptotic genes and many pluripotency genes were downregulated. Most lineage commitment genes other than ectoderm markers have been upregulated after Dax1KO (Figure 6(a)). We then validated the expression of key genes by qRT-PCR, and the results were consistent with our RNA-seq data (Figure 6(b)).

## 3. Discussion

Reduced expression of Dax1 has been reported to induce conventional ESCs differentiation, suggesting the essential role of Dax1 in self-renewal [5, 6]. While the phenotypes observed in these studies were based on the transient suppression of Dax1. In this study, we established a stable Dax1KO cell line using the CRISPR/Cas9 system and found that Dax1 knockout caused ESCs differentiation and death. Therefore, our results confirmed and extended the previous study that continuous expression of Dax1 is necessary to maintain the pluripotency of ESCs in LIF/serum.

Dax1 appears to be critical for the core pluripotency circuitry of conventional ESCs [1, 2, 18], but the effect of the loss of function of Dax1 on the pluripotency-associated transcription factor network was not well analyzed. The regulatory transcription networks of conventional ESCs can be subdivided into distinct units based on physical bindings of protein and DNA. Core module includes genes that are mainly related to developmental and transcription-associated processes, and the Myc module contains targets which are predominantly involved in cellular metabolism, cell cycle, and protein synthesis pathways. The main role of the PRC module is to trimethylate H3K27 and cause target gene silencing [15, 19, 20]. Dax1 was regarded as a pluripotency-associated factor, which belonged to the core module. Here, we reported that Dax1KO not only caused a significant reduction in the activity of the core module but also reduced the activity of the Myc module and activating the expression of developmental genes silenced by PRC2. This suggested a functional link among the three relatively independent modules, and in which Dax1 might play an important role. Previous studies revealed that c-Myc can upregulate the transcription of the Polycomb PRC2 complex [21], and there is a transcriptional regulatory relationship between the core module members Nac1 and c-Myc [19]. Further study will be required to reveal the molecular functions of Dax1 on different modules of the transcriptional regulatory network.

It is currently believed that pluripotency is not a monolithic entity but rather comprises a spectrum of different cellular states [9]. Dax1 is expressed at a high level in undifferentiated ESCs but not in EpiSCs, suggested that the expression of Dax1 is affected by different stages of pluripotency. However, it is not clear whether the expression of Dax1 in 2i/LIF-ESCs is different from the conventional ESCs. We reported that the expression level of Dax1 in 2i/LIF-ESCs was significantly reduced, and the regulation occurred at the transcription level. Dax1 expression has been shown to be upregulated by the LIF/Stat3 pathway as well as by Oct4 [22], Nanog, and Nr5a2 [23]. However, these positive regulators of Dax1 were not significantly reduced in 2i/LIF, so they cannot be used to explain the differential expression of Dax1 under the two culture conditions. Under the 2i/LIF culture conditions, Gsk3 (a negative regulator of Wnt effector *β*-catenin) and MAPK were selectively inhibited. Early studies showed that Dax1 expression was controlled by Wnt signaling in the gonad [24], but BIO (a pharmacological inhibitor of GSK-3) treatment did not significantly modulate Dax1 expression in ESCs cultured in the presence of LIF. The MAPK/ERK pathway can regulate protein expression levels through phosphorylation, leading to the stability of c-Myc protein and the degradation of Klf2 protein, respectively. But whether MAPK/ERK regulates the expression of Dax1 has not been reported. Overall, the molecular basis of transcriptional suppression of Dax1 in ground state ESCs is not currently known.

Accumulating evidences suggest that the 2i/LIF ESCs represent the naive epiblast cells of the inner cell mass (ICM) or even earlier stages, while conventional ESCs possibly reflect later stages. And as 2i/LIF and LIF/serum ESCs are readily interconvertible, they provide a unique and accessible in vitro model to explore the regulation of the pre- to postimplantation phase of early embryonic development. In recent years, it has become clear that the transcriptome, epigenome, and methylome of 2i/LIF and LIF/serum ESCs are markedly different. However, the characterization of individual genes in different pluripotency states research is still very limited. We reported that the colonies of the Dax1KO cell line could be passaged at least 30 times in 2i/LIF (data not shown) with no change in morphology compared with control cells, which is completely different from that in LIF/serum. This suggested that the two pluripotency states have different requirements for Dax1, and Dax1 is dispensable for the self-renewal of 2i/LIF-ESCs.

To provide more insights into Dax1 function, we detected whether Dax1KO ESCs could be adapted to LIF/serum supplemented with the 2i inhibitors, PD0325901 (PD) and/or CHIR99021 (CH). Dax1KO ESCs formed very few compact colonies in LIF/serum, while formed irregular flat colonies in LIF/serum (LS)+PD. In LS+CH, the number of compact colonies was significantly higher than that in LIF/serum, but there were still some differentiated colonies. Only in the LS+2i, Dax1KO ESCs could form compact colonies similar to the normal control (Figure S2a). The cell count results showed that both PD and CH could promote the proliferation of Dax1KO ESCs, and the proliferation effect of CH was more obvious (Figure S2b). The above results suggested that Dax1 might not directly mediate the effects of ERK or Wnt pathways.

Based on the previous ChIPseq results [1, 25], the target genes under the transcriptional regulation of Dax1 were deduced. These putative target genes showed less activation/repression in 2i/LIF compared to LIF/serum after Dax1KO (Figure S3). GO analysis showed that 267 overlap DEGs (>2 fold, *P* < 0.05) of Dax1KO in 2i/LIF and LIF/serum (Figure 4(b), Figure S4a) were enriched in gene sets including “Development process” and “Cell proliferation” (Figure S4b). While only a few overlap DEGs are putative target genes of Dax1 (Figure S4c). This suggests that in addition to transcriptional regulation, Dax1 may also affect gene expression through other ways.

The core module was identified in conventional ESCs, but genome-wide ChIP-Seq localization studies for these proteins have not been performed in 2i/LIF-ESCs. Dax1KO caused a significant reduction in the activity of the core module in 2i/LIF-ESCs as well as in conventional ESCs, but it was not accompanied by a self-renewal impaired phenotype in 2i/LIF. It is suggested that the core module identified in conventional ESCs is either not important or the composition of the module is different in 2i/LIF.

In conclusion, the results of the present study demonstrated that the cell phenotype and molecular phenotype produced by Dax1KO in LIF/serum and 2i/LIF cultured ESCs are significantly different. This may reflect the different cell fate determination mechanisms of conventional ESCs and ground state ESCs. Dax1 is an oncogene, but its expression pattern in cancer progression has shown discrepancy among different types of cancers. Our research not only provides clues for understanding the molecular regulation mechanism at different stages of early embryonic development but also contributes to a better understanding of Dax1's role in different tumors.

## 4. Materials and Methods

### 4.1. Cell Culture

Mouse ESCs were cultured on 0.2% gelatin-coated plates in the indicated medium. LIF/serum culture: DMEM supplemented with 15% FBS, 2 mM GlutaMAX, 1% MEM nonessential amino acids, 0.1 mM *β*-mercaptoethanol (all from Invitrogen), and 10 ng/ml LIF (Millipore). 2i/LIF culture: N2B27 medium was supplemented with 1 *μ*M PD0325901, 3 *μ*M CHIR99021 (Selleck), and 10 ng/ml LIF [26]. 293FT cells were maintained in DMEM supplemented with 10% FBS, 2 mM GlutaMAX, and 1% MEM nonessential amino acids.

### 4.2. Plasmid Construction

For the CRISPR Cas9-induced Dax1 knockout (KO), single guide RNA targeting Dax1 was designed around the Translation Start Codon. The following guide sequence (5′-acaggagcctcaggccatggcgggtgaggaccacc-3′) was cloned in the pLentiCas9-Zeocin vector. The full-length open reading frames of Dax1 were PCR-amplified from mouse ESC cDNA using primers (Forward: GAATTCGGATCCACCATGGCGGGTGAGGACCACC, Reverse: GGATCCGAATTCTCACAGCTTTGCACAGAGCA). The amplified ORF was subsequently cloned into pGEM-T Easy (Promega) for sequence verification and, then, subcloned into pPGK.2AP.

### 4.3. Lentiviral Production

Lentiviral vector, pSPAX2, and pMD2G were cotransfected into 293FT cells by calcium phosphate transfection as previously described [3]. In brief, 12.5 *μ*g plasmids in total were mixed with 50 *μ*l CaCl_2_ (2.5 mM) and diluted with TE buffer to a final volume of 0.5 ml. The mixture was added dropwise to 0.5 ml 2 × HeBS and mixed. One minute later, the solution was mixed and added dropwise into the media. 8-10 hours later, the medium was changed, and the virus was collected after a subsequent 48-72 h cultivation.

### 4.4. Generation of Dax1KO-ESCs

ESCs were trypsinized and infected in suspension by the Dax1KO lentiviral supernatant along with polybrene (4 *μ*g/ml, sigma). Two days later, these cells were replated at 1 × 10^3^ cells per 6-well plates and cultured with 15 *μ*g/ml zeocin (Invitrogen) for 7 days. The resulting colonies were picked up, expanded, and identified by immunoblotting.

### 4.5. Plasmid Transfection

ESCs were transfected with plasmid DNA using Lipofectamine2000 (Invitrogen) according to the manufacturer's instructions. Two days later, cells were cultured in the presence of 1 *μ*g/ml puromycin (Invitrogen) to gain stable transfection.

### 4.6. In Vitro and In Vivo Differentiation of ESCs

EB formation was performed in petri dishes in FBS medium without LIF according to a previously described protocol^2^.

### 4.7. Cell Proliferation and Colony Formation Assay

Cell proliferation and colony formation assay was derived using a previously described protocol^2^. For proliferation assay, cells were plated in gelatin-coated 12-well plates. After 5 days, viable cells were determined. For colony formation assay, cells were plated in gelatin-coated 24-well plates. After 6 days, AP positive colonies were measured. Colonies were scored in three categories: undifferentiated, mixed (partially differentiated), and differentiated.

### 4.8. Real-Time PCR Analysis

Total RNA was isolated with TRIzol reagent (Invitrogen, Cat: 15596026) as previously described. First-strand cDNA was synthesized from 1 *μ*g of total RNA with oligo-dT primer using the Transcriptor First Strand cDNA Synthesis Kit (Roche), and diluted ten-folds with water. Real-time PCR reaction was performed with the FastStart Essential DNA Green Master (Roche) using the LightCycler 96 System (Roche). The qPCR primers are listed in Supplementary Table 2.

### 4.9. Protein Extraction and Western Blot

Protein extraction and Western blot were performed with anti-Dax1 (Active Motif, #39983) according to a previously described protocol^2^.

### 4.10. FACS Analysis

For apoptotic assay, cells were stained with Annexin V-APC (BD Biosciences) and propidium iodide for 10 minutes at 4°C. For cell cycle assay, cells were fixed with 70% ethanol overnight at -20°C and, then, treated with RNase and propidium iodide (Beyotime) for 20 minutes at 37°C. Cells were analyzed with Novocyte (ACEA).

### 4.11. RNA-Seq and Data Analysis

Total RNA was extracted using Trizol Reagent according to the manufacturer's manual. Two RNA samples of every indicated cell lines were processed by Genminix Informatic Ltd. (Shanghai, China) for mRNA sequencing on Illumina Hiseq platform (Illumina) with 6Gbps. Differential gene expression (DEG) analysis was performed using the DESeq2 (v1.14.1) R statistical programming software package (Love et al., 2014). Threshold for DEGs was set to *P* value < 0.05 and absolute fold change >1.2 or 2. The DEGs are listed in Supplementary Table 3. Gene Ontology enrichment analysis of DEGs was performed using the Enrichr website (Supplementary Table 4) [27].

### 4.12. Statistical Analysis

Statistical analysis was performed using the Statistical Package for Social Science. Student's *t*-test was used to analyze statistical differences. Data in the figures were expressed as mean ± s.d., and *P* ≤ 0.05 was considered significant. Each experiment was performed at least three times.

## Data Availability

RNA-seq data that support the findings of this study have been deposited in the Gene Expression Omnibus (GEO) under the accession numbers GSE168423. Source data for Figures 4–6 have been provided as Supplementary Table 3 and Supplementary Table 4.

## Supplementary Materials

Supplementary Materials Figure S1: Dax1 expression pattern during early embryonic development. Figure S2: Dax1KO ESCs were cultured in LIF/serum supplemented with MEK inhibitor PD0325901 and/or GSK3 inhibitor CHIR99021. Figure S3: the differential expression of Dax1 target genes in 2i/LIF and LIF/serum. Figure S4: the analysis of overlap DEGs caused by Dax1KO in 2i/LIF and LIF/serum. Table S1: Previously reported loss-of-function analyses of Dax1 in ESCs. Table S2: primers for RT-PCR analysis. Table S3: DEGs in RNA-seq analysis (PDF format). Table S4: GO Annotation Enrichment Analysis (PDF format).
