# Supplementary material for: Pluripotency-State-Dependent Role of Dax1 in Embryonic Stem Cells Self-Renewal
Source: Stem Cells Int. 2021 Jul 10;2021:5522723. doi: 10.1155/2021/5522723 (PMC8286181; doi:10.1155/2021/5522723)
Supplement: Supplementary Materials — Figure S1: Dax1 expression pattern during early embryonic development. Figure S2: Dax1KO ESCs were cultured in LIF/serum supplemented with MEK inhibitor PD0325901 and/or GSK3 inhibitor CHIR99021. Figure S3: the differential expression of Dax1 target genes in 2i/LIF and LIF/serum. Figure S4: the analysis of overlap DEGs caused by Dax1KO in 2i/LIF and LIF/serum. Table S1: Previously reported loss-of-function analyses of Dax1 in ESCs. Table S2: primers for RT-PCR analysis. Table S3: DEGs in RNA-seq analysis (PDF format). Table S4: GO Annotation Enrichment Analysis (PDF format). [file 5522723.f1.docx]

**List of supplemental information**

**Supplemental Figures**

Figure S1. Dax1 expression pattern during early embryonic development.

Figure S2. Dax1KO ESCs were cultured in LIF/serum supplemented with MEK inhibitor PD0325901 and/or GSK3 inhibitor CHIR99021.

Figure S3. The differential expression of Dax1 target genes in 2i/LIF and LIF/serum.

Figure S4. The analysis of overlap DEGs caused by Dax1KO in 2i/LIF and LIF/serum.

**Supplemental Tables**

Table S1. Previously reported loss-of-function analyses of Dax1 in ESCs

Table S2. Primers for RT-PCR analysis

Table S3. DEGs in RNA-seq analysis (PDF format).

Table S4. GO Annotation Enrichment Analysis (PDF format).

**
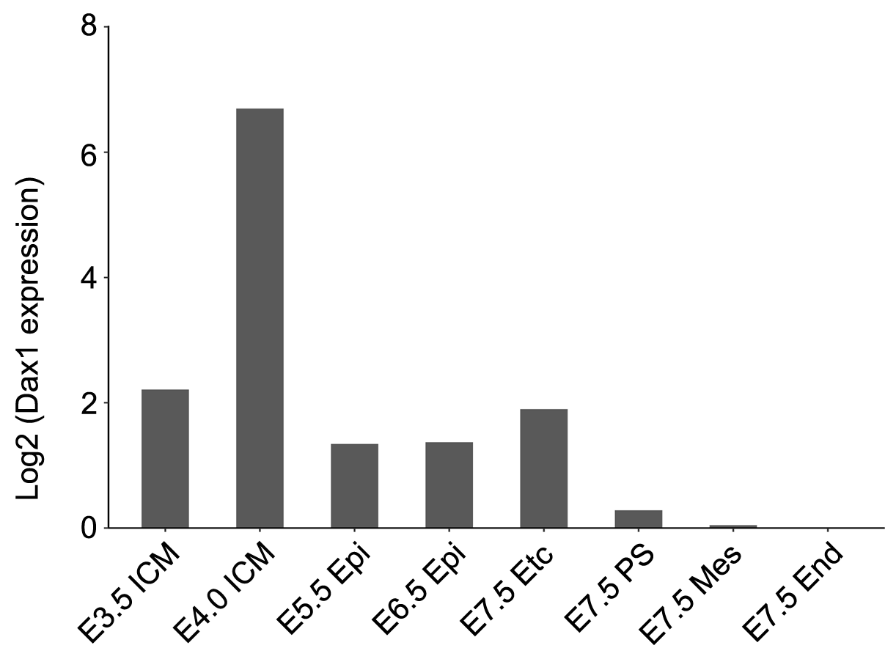
**

**Figure S1. Dax1 expression pattern during early embryonic development.**


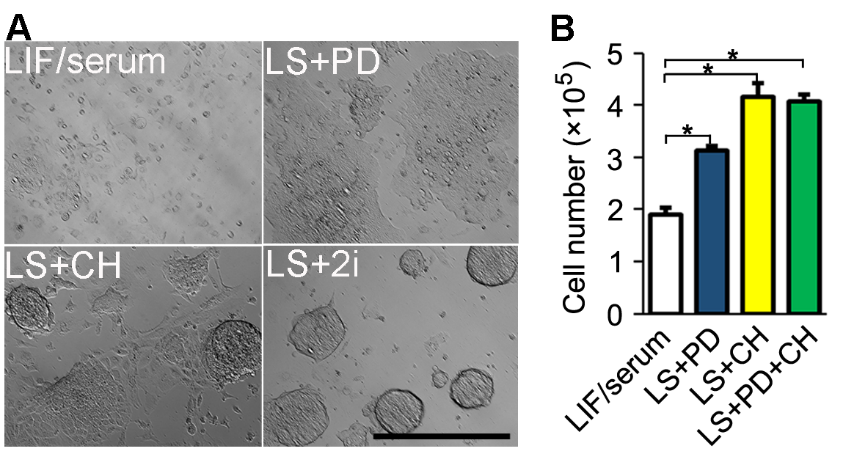


**Figure S2. Dax1KO ESCs were cultured in LIF/serum supplemented with MEK inhibitor PD0325901 and/or GSK3 inhibitor CHIR99021.**

1. Representative images of Dax1KO ESCs in the indicated cultures for five days (Scale bar, 100 μm)
2. Dax1KO ESCs (1000 cells/cm^2^ in 12-well plates) were cultured for five days in the indicated cultures and cell numbers were counted. (LS = LIF/serum, PD = MEK inhibitor PD0325901, CH=GSK3 inhibitor CHIR99021, 2i=PD+CH )


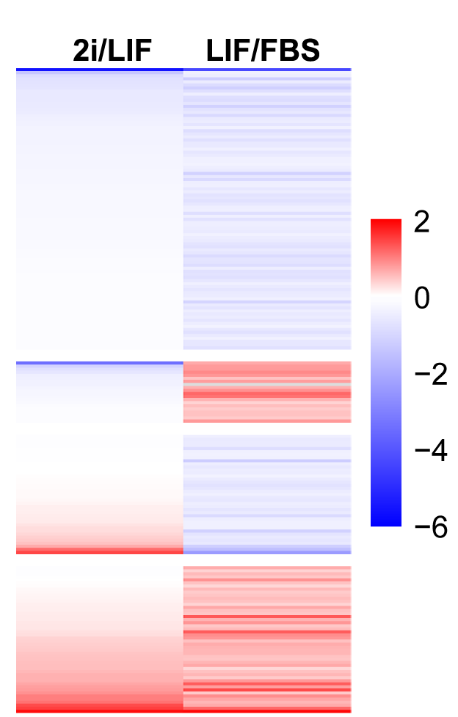


**Figure S3. The differential expression of Dax1 target genes in 2i/LIF and LIF/serum.**

Heatmap showing the geometric p values calculated by gene set overlap analysis between gene sets from ChIP-seq and DEGs (Dax1KO compared to wild-type mESCs cultured in 2i/LIF and LIF/serum). The red cells correspond to up-regulated DEGs, blue cells correspond to down-regulated DEGs. Color intensity is proportional to log2 (p-value).


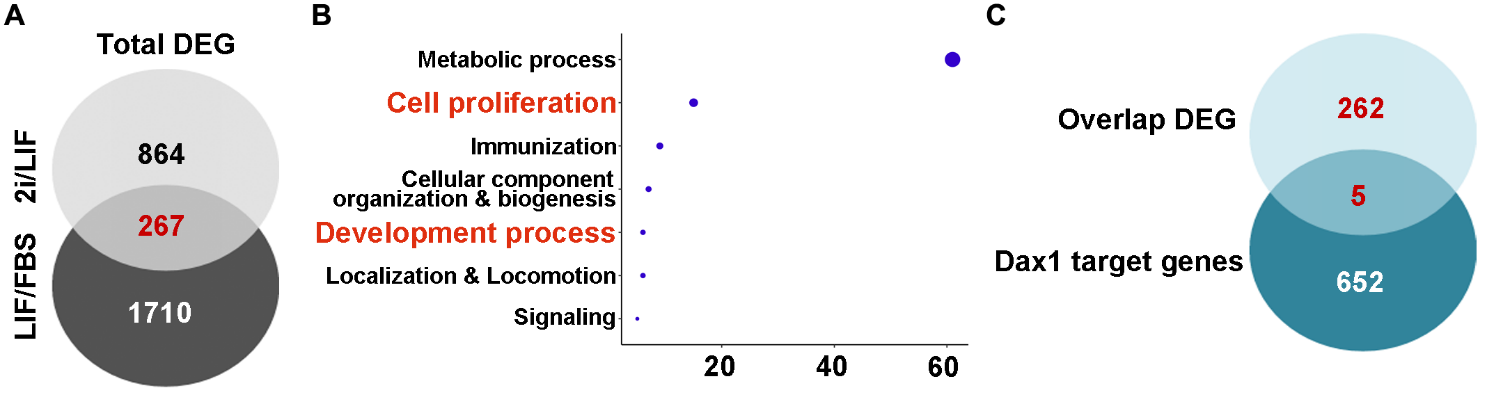


**Figure S4. The analysis of overlap DEGs of Dax1KO in 2i/LIF and LIF/serum**

1. Venn diagrams represent overlapped total DEGs in Dax1 KO cells between 2i/LIF and LIF/serum medium (>2 fold, P<0.05).
2. GO analysis of 267 overlap DEGs of Dax1KO in 2i/LIF and LIF/serum (>2 fold, P<0.05).
3. Venn diagrams represent the overlap between 267 overlapped total DEGs and Dax1 target genes.

**Supplemental Table 1. Previously reported loss-of-function analyses of Dax1 in ESCs**

| Study | Cell culture | Methods | Time of analysis | Phenotype |
| --- | --- | --- | --- | --- |
| (Yu et al., 1998)[[1](#_ENREF_1)] | 20% Serum + LIF | 1. A standard strategy for deletion of Dax1 exon 1; 2. Cre-mediated disruption exon 2 of Dax1 | transiently | Failed to generate undifferentiated ESC |
| (Niakan et al., 2006)[[2](#_ENREF_2)] | 15% Serum + LIF | 1. Conditional knockout(KO) by deletion of Dax1 exon 1 2. Knockdown(KD), siRNA | Transiently(2days) | Endoderm differentiation |
| (Wang et al., 2006)[[3](#_ENREF_3)] | 15% Serum + LIF | KD, retroviral shRNA | Transiently(4days) | Loss of pluripotency, multilineage differentiation |
| (Khalfallah et al., 2009)[[4](#_ENREF_4)] | 10% Serum +LIF | KD, siRNA | Transiently(4days) | Multilineage differentiation, decreased viability andproliferation |
| (Zhang et al., 2014)[[5](#_ENREF_5)] | 5%Serum+15%KSR + LIF | Stably KD, retroviral shRNA | Continuously propagated for at least 30 passages | Increased differentiation propensity and reduced self-renewal efficiency |
| (Fujii et al., 2015) [[6](#_ENREF_6)] | 10%Serum +LIF, on MEF | 1. Inducible KO by deletion of Dax1 exon 2 | Continued self-renewal with slow proliferation ratio | Dispensable for maintaining pluripotency |

**Supplemental References**

1. Yu RN, M Ito, TL Saunders, SA Camper and JL Jameson. (1998). Role of Ahch in gonadal development and gametogenesis. Nat Genet 20:353-7.

2. Niakan KK, EC Davis, RC Clipsham, M Jiang, DB Dehart, KK Sulik and ER McCabe. (2006). Novel role for the orphan nuclear receptor Dax1 in embryogenesis, different from steroidogenesis. Mol Genet Metab 88:261-71.

3. Wang J, S Rao, J Chu, X Shen, DN Levasseur, TW Theunissen and SH Orkin. (2006). A protein interaction network for pluripotency of embryonic stem cells. Nature 444:364-8.

4. Khalfallah O, M Rouleau, P Barbry, B Bardoni and E Lalli. (2009). Dax-1 knockdown in mouse embryonic stem cells induces loss of pluripotency and multilineage differentiation. Stem Cells 27:1529-37.

5. Zhang J, G Liu, Y Ruan, J Wang, K Zhao, Y Wan, B Liu, H Zheng, T Peng, W Wu, P He, FQ Hu and R Jian. (2014). Dax1 and Nanog act in parallel to stabilize mouse embryonic stem cells and induced pluripotency. Nat Commun 5:5042.

6. Fujii S, S Nishikawa-Torikai, Y Futatsugi, Y Toyooka, M Yamane, S Ohtsuka and H Niwa. (2015). Nr0b1 is a negative regulator of Zscan4c in mouse embryonic stem cells. Sci Rep 5:9146.

**Supplemental Table2. Primers for qRT-PCR**

| Gene | Primer Sequence(5’-3’) | |
| --- | --- | --- |
|  | Forward | Reverse |
| GAPDH | AGGTCGGTGTGAACGGATTTG | TGTAGACCATGTAGTTGAGGTCA |
| c-Myc | TCACCAGCACAACTACGCCG | CAGGATGTAGGCGGTGGCTT |
| n-Myc | CTGAGCTGGTGAAGAACGAG | TCGATCTTCTTTAGCAACTGC |
| Ntn1 | CGGGAGAAGAAGGGCAAGTG | CCCTGACGGCCAAGTCCAAG |
| Bmf | GAACCCCAGAGACTCTTTTAC | TGCTGGTGTTGTTGCGTATG |
| Bbc3 | CTGGAGGGTCATGTACAATC | CTACATGGTGCAGAAAAAGTC |
| Bcl2l13 | GCAGTGGCTGTTGGAGTTGC | GCTGCTGGCAGTCTAAGTTC |
| Dax1 | TCCTGTACCGCAGCTATGTG | ATCTGGAAGCAGGGCAAGTA |
| Nanog | TCTTCCTGGTCCCCACAGTTT | GCAAGAATAGTTCTCGGGATGAA |
| Klf2 | AAGGATGCGGCTGGAAGTTC | CTGTGACCTGTGTGCTTTCG |
| Nr5A2 | GAGCTCTTGATTCTCGATCACA | AACTCCCGCTGATCGAACTGAA |
| Id1 | GATCTCTGGGAAAGACACTAC | GGGTGAGGCTCTGTTGATCAA |
| Gbx2 | GCTACAGCCACACCAGAAGATA | CTGAGATCCAGTCTAGATAGAG |
| Sox1 | CCAAGATGCACAACTCGGAGATCA | TAATCCGGGTGTTCCTTCATGTGC |
| Brachyury | CTGCGCTTCAAGGAGCTAAC | CCAGGCCTGACACATTTACC |
| Flk1 | TTTGGCAAATACAACCCTTCAGA | GCAGAAGATACTGTCACCACC |
| Gata6 | AAAGCTTGCTCCGGTAACAG | TTCTCCCACTGCAGACATCA |
| Sox17 | GTGGACCGCACGGAATTCGAA | GCAATAGTAGACCGCTGAGCTA |
| Hand1 | TGAACTCAAAAAGACGGATGG | CTTTAATCCTCTTCTCGCCG |
